# Evaluation of Reliability of the Recomputed Nutrient Intake Data in the National Heart, Lung, and Blood Institute Twin Study

**DOI:** 10.3390/nu11010109

**Published:** 2019-01-08

**Authors:** Yecheng Yao, Sheng-Bo Chen, Gangqiang Ding, Jun Dai

**Affiliations:** 1 National Institute for Nutrition and Health, Chinese Center for Disease Control and Prevention, Beijing 100050, China; yaoyc@ninh.chinacdc.cn (Y.Y.); dinggq@chinacdc.cn (G.D.); 2 Educational Technology Doctoral Program, Department of Educational Studies, College of Education and Human Ecology, The Ohio State University, Columbus, OH 43210, USA; chen.4880@buckeyemail.osu.edu; 3 Department of Public Health, College of Health Sciences, Des Moines University, Des Moines, IA 50312, USA

**Keywords:** twin study, nutrient, food composition database

## Abstract

The nutrient intake dataset is crucial in epidemiological studies. The latest version of the food composition database includes more types of nutrients than previous ones and can be used to obtain data on nutrient intake that could not be estimated before. Usual food consumption data were collected among 910 twins between 1969 and 1973 through dietary history interviews, and then used to calculate intake of eight types of nutrients (energy intake, carbohydrate, protein, cholesterol, total fat, and saturated, monounsaturated, and polyunsaturated fatty acids) in the National Heart, Lung, and Blood Institute Twin Study. We recalculated intakes using the food composition database updated in 2008. Several different statistical methods were used to evaluate the validity and the reliability of the recalculated intake data. Intra-class correlation coefficients between recalculated and original intake values were above 0.99 for all nutrients. *R*^2^ values for regression models were above 0.90 for all nutrients except polyunsaturated fatty acids (*R*^2^ = 0.63). In Bland–Altman plots, the percentage of scattering points that outlay the mean plus or minus two standard deviations lines was less than 5% for all nutrients. The arithmetic mean percentage of quintile agreement was 78.5% and that of the extreme quintile disagreement was 0.1% for all nutrients between the two datasets. Recalculated nutrient intake data is in strong agreement with the original one, supporting the reliability of the recalculated data. It is also implied that recalculation is a cost-efficient approach to obtain the intake of nutrients unavailable at baseline.

## 1. Introduction

Data on food consumption, nutrient intake, and dietary compounds is essential to examine the role of diet in disease. In epidemiological studies, data on food consumption can be collected through the means of dietary assessment methodology, such as duplicate diet approach, dietary records, 24-h dietary recall, food frequency, brief dietary assessment instruments, diet history, or blended instruments [1,2]. Data on nutrient intake and other dietary compounds is conventionally calculated through the joint use of food consumption data and the food composition database.

A food composition database is composed of nutrients and dietary compounds and their amount in a defined amount of an edible food item, i.e., 100 g and/or one serving. It is updated and expanded continuously [3]. Commonly, abaseline nutrient dataset contains a limited number of nutrients in longitudinal studies, as the earlier versions of the database contained far fewer nutrients and dietary compounds than updated versions of the database. With advancement in both knowledge and role of nutrients and dietary compounds in relation to disease, the early nutrient database with its limited number of nutrients has become a barrier to investigate the early dietary intake in terms of current nutrient/disease interest [4]. This barrier can be overcome cost-effectively by creating a recalculated nutrient dataset from existing baseline food consumption data and the updated version of the food composition database.

The National Heart, Lung, and Blood Institute (NHLBI) Twin Study is a 49-year longitudinal study [5,6]. This study is gradually gaining importance in nutritional epidemiological studies [7]. However, when research interest lies in the 49-year follow-up outcomes in relation to nutrition, it would be extremely costly and time-consuming to establish a new longitudinal twin study for new dietary data collection [8,9]. By contrast, applying an updated food composition database that contains more types of nutrients and compounds, recalculation of nutrient intake from the early dietary intake can avoid intensive labor, save long-term follow-up time, and save considerable costs in large-scale population studies [10,11,12].

It is important to evaluate the validity and the reliability of recalculated data for epidemiologic studies [4]. The validity and reliability of dietary data was defined as the ability to group and rank participants based on individuals’ diet among a population [13,14]. The purpose of this study is to assess the validity and reliability of the eight recalculated types of nutrients in the NHLBI Twin Study.

## 2. Subjects and Methods

### 2.1. Study Population

As described previously [7,15,16,17,18], the NHLBI Twin Study is the longest longitudinal twin study of genetic, dietary, and other environmental factors in cardiovascular disease. At the baseline examination (1969–1973), this study enrolled 514 middle-aged, white, male, veteran twin pairs (1028 men, 254 monozygotic and 260 dizygotic twin pairs), who were born between 1917 and 1927 and were 42–55 years of age. Zygosity was ascertained using eight red blood cell antigen groups (serotyping 22 erythrocyte antigens) in the 1960s and a variable number of tandem repeat DNA markers in the 1980s. The study protocol was approved by the Institutional Review Board at each examination site, and all twins gave written informed consent. Fifty-nine twin pairs were excluded for not providing food consumption data at baseline. Finally, a total of 455 twin pairs (234 monozygotic pairs and 221 dizygotic pairs) were included for this study. This study was approved by the Institutional Review Board at Indiana University-Bloomington (Protocol number: 0903000157).

### 2.2. Baseline Food Consumption Data and the NHLBI Nutrient Dataset

The food consumption data at baseline was collected by nutritionists through in-person interviews by means of standardized nutritionist-administered, cross-checked, dietary history interviews adapted from Burke’s method [19]. All questions on the dietary history interview questionnaire were related to usual meals and eating habits. This questionnaire was validated in the Framingham Study [13,20]. Detailed information about this NHLBI Twin Study questionnaire was previously published [5,6]. One questionnaire was used at baseline. Food intake data were collected by this questionnaire concerning the following: quantitative evaluation of the frequency of the American diet consumed in a given day or over a period of one week; double-checking the accuracy of responses to the quantitative information; and, characterizing meal and snack habits regarding time and frequency [5]. The original baseline nutrient intake data were derived through the joint use of food consumption data and the NHLBI Twin Study food composition table (i.e., Table 3.3.0.1) developed in the early 1970s [5,6]. This nutrient dataset contains the intake of nutrients including: total energy intake (kcal/day), total carbohydrate (g/day), simple carbohydrate (g/day), complex carbohydrate (g/day), protein (g/day), cholesterol (mg/day), total fat (g/day), saturated fatty acids (g/day) (SFA), monounsaturated fatty acids (g/day) (MUFA), and polyunsaturated fatty acids (g/day) (PUFA). In this reported study, we focused on eight nutrients: total energy intake, total carbohydrate, protein, cholesterol, total fat, SFA, MUFA, and PUFA.

### 2.3. Recalculation of Nutrients Intakes

The Standard Reference 21 (SR21), a large food composition database, was released by the United States Department of Agriculture (USDA) in 2008 [21]. Nutrient intake data were recalculated through joint use of the baseline food consumption data from the NHLBI Twin Study and the SR21. Each food item in the dietary history interview questionnaire (excluding items for double-checking the accuracy of responses) was matched to a corresponding food item in SR21 for the content of nutrients (i.e., protein, total fat, and total carbohydrate) in the NHLBI Twin Study food composition Table 3.3.0.1 (the most similar matching) (Table 1) [5]. A senior registered dietitian, who was familiar with food and diets in the United States since late 1960s, identified food items from SR21 that corresponded to those in the NHLBI Twin Study. The combined food items that contained more than one food item were split into ingredients that could be matched for those in SR21 by considering the most similar nutrient composition. A statistical program was developed using statistical analysis software (SAS^®^ 9.2, SAS Institute Inc., Cary, NC, USA) for the recalculation of nutrient intake.

### 2.4. Statistical Analyses

Analyses of correlation (including intra-class, Pearson’s correlations), linear regression, quintile agreement, and extreme quintile disagreement were performed [22,23,24,25]. The intra-class correlation coefficients (ICC) were used to assess the similarity of the recalculated and original data. Pearson’s correlations, regression, and quintile agreement or extreme quintile disagreement of the continuous data were calculated because these measures gave valuable basic information about the recalculated and original data. We performed a simple linear regression model, in which recalculated nutrient intake was the dependent variable and original nutrient intake was the independent variable. Graphic techniques (regression plot and Bland–Altman plots) were also used to illustrate the relation between original and recalculated data. Bland–Altman plots of differences in nutrient intakes (recalculated mean values minus the corresponding original ones) against the average of these two values are performed in this study. We also categorized twins into quintiles according to the recalculated and the original nutrient intake datasets, separately. The percentage of twins classified into the same quintile using the two sets of nutrient data was calculated as the quintile agreement [26]. The percentage of twins who were classified into one extreme quintile (the top or the bottom quintile) using the original dataset while into the opposite extreme quintile (the bottom or the top quintile) using the recalculated one was calculated as the extreme quintile disagreement (opposite extreme quintile). Statistical software (SAS 9.2) was used. A *p*-value < 0.05 was considered statistically significant (two-sided).

## 3. Results

### 3.1. Univariate Analyses

The recalculated mean intake values were very similar to those in the original database for most nutrients. The percent mean differences of nutrient intake between the recalculated and the original values were less than 10% for six nutrients (total energy intake, carbohydrate, protein, total fat, SFA, and cholesterol) and greater than 35% for MUFA and PUFA (Table 2).

### 3.2. Correlation and Regression Analyses

Intra-class correlation (ICC) coefficients were above 0.99 for all nutrients. Pearson’s correlations coefficients were above 0.95 for seven nutrients (total energy intake, carbohydrate, protein, total fat, SFA, MUFA, and cholesterol) and *p*-value < 0.05 for all nutrients (Table 3). The *R*-square (*R*^2^) values from the regression model were above 0.90 for the same seven nutrients, except for PUFA (Table 3).

### 3.3. Graphic Analyses

**Figure 1:**
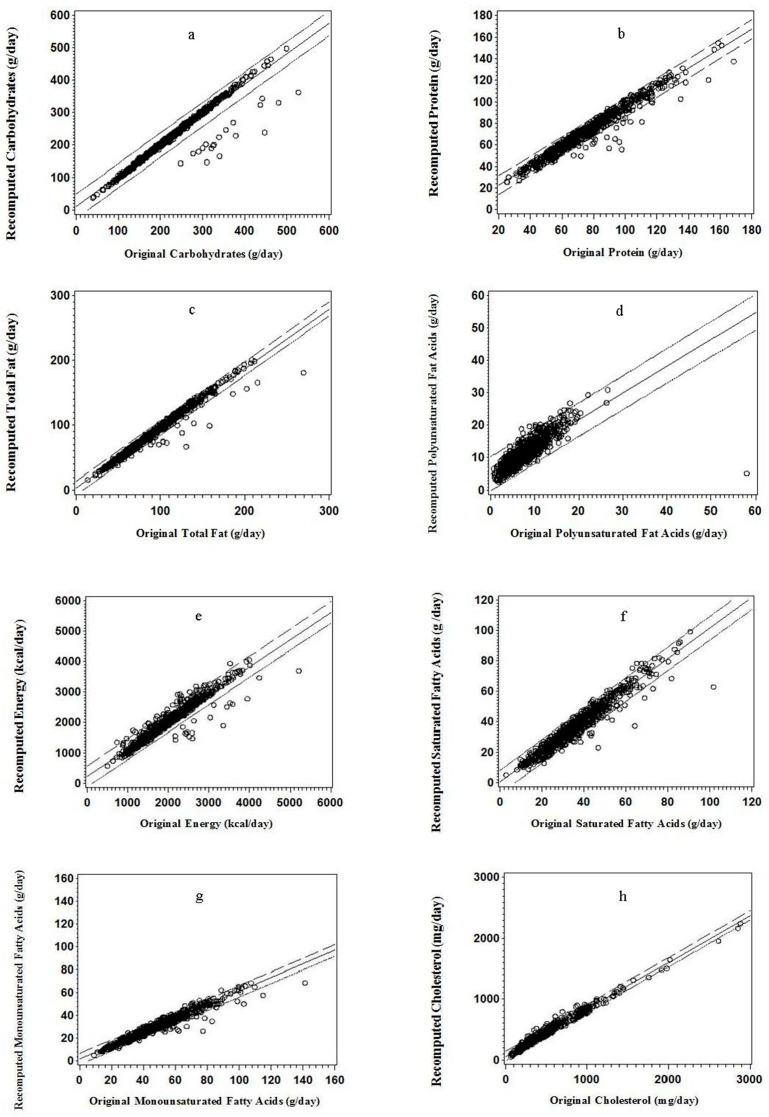
Regression plots for the recalculated value versus the original value: (**a**) carbohydrate; (**b**) protein; (**c**) total fat; (**d**) polyunsaturated fatty acids; (**e**) energy; (**f**) saturated fatty acids; (**g**) monounsaturated fatty acids; and (**h**) cholesterol (*n* = 910).

For protein, carbohydrate, total fat, and PUFA, less than 5% of point scatter was above or below the 95% confidence intervals (95% CI) of the regression line; and the data points tended to scatter more below the 95% CI of the regression line than above it except those for PUFA (Figure 1a–d). Similar regression plot pattern was found for energy intake, SFA, MUFA, and cholesterol (Figure 1e–h).

**Figure 2:**
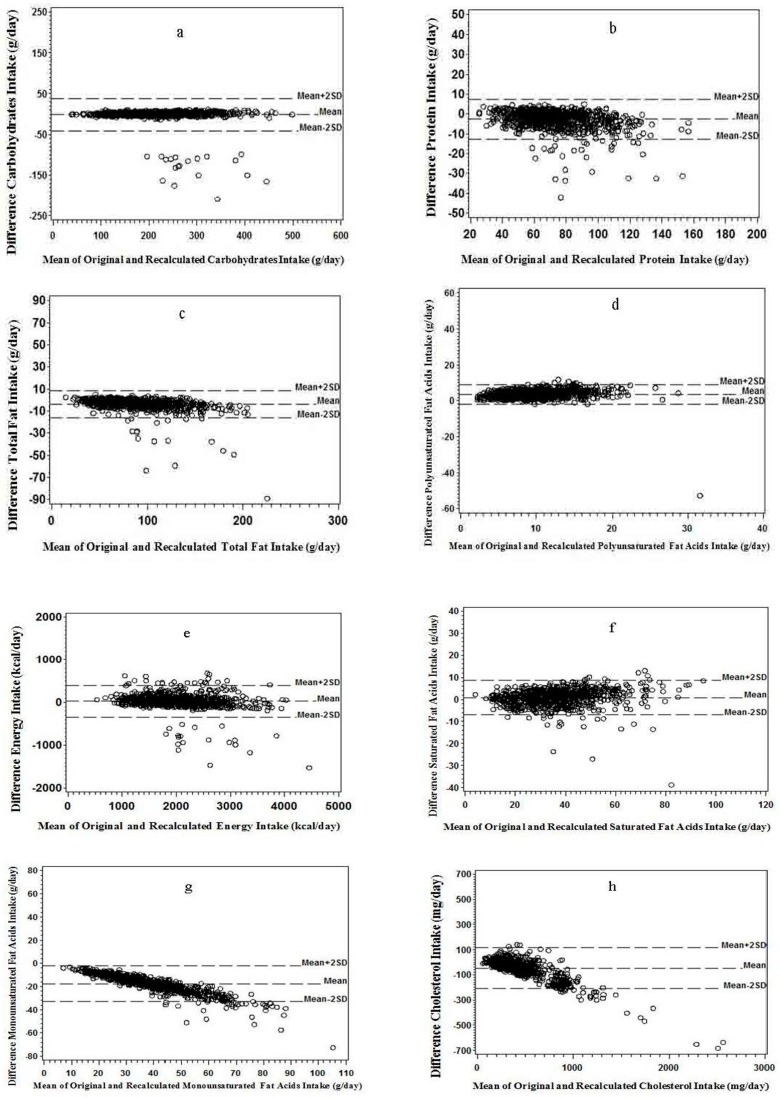
Bland–Altman plots for differences between versus mean of the recalculated and the original value for: (**a**) carbohydrate; (**b**) protein; (**c**) total fat; and (**d**) polyunsaturated fatty acids. Bland–Altman plots for differences between versus mean of the recalculated and the original value for: (**e**) energy; (**f**) saturated fatty acids; (**g**) monounsaturated fatty acids; and (**h**) cholesterol (*n* = 910).

Figure 2a–d illustrates that less than 5% of points were scattered above the line of mean plus two standard deviations (mean + 2SD) or below the mean minus two standard deviation (mean − 2SD) lines. A similar trend was observed in the Bland–Altman plots for energy intake and SFA, and slight downward trends were observed for MUFA and cholesterol, indicating that a weaker consistency may appear at greater intake values (Figure 2e–h). The point scatter tended to be more below the mean − 2SD line of than above the mean + 2SD line for all nutrients except PUFA (Figure 2a–h).

### 3.4. Quintile Agreement and Extreme Quintile Disagreement

The mean percentage for the same quintile agreement was 78.5%, whereas the mean percentage of the extreme quintile disagreement was 0.1% (Table 4). The percentage of the same quintile agreement was greater than 75% for all nutrients except PUFA (51.2% for PUFA) (Table 4). The percentage of the extreme quintile disagreement was below 0.1% for all nutrients (Table 4).

## 4. Discussion

We found that the recalculated and original intakes of eight types of nutrients were highly correlated. In Bland–Altman plots, the percentage of scattering points outlying the mean ± 2SD lines was low. The percentage of extreme quintile disagreement was extremely low for all nutrients, while the percentage of the same quintile agreement was very high. In general, our findings demonstrate a strong agreement between recalculated and raw data, supporting the validity and reliability of the recalculated nutrient intake data to group and rank participants. Taken together, our study supports the validity of the recalculated nutrient intake data; that is, expansion of the original NHLBI Twin Study nutrient dataset can be feasible and practical.

Using agreement analyses, Bazzano et al. reported the validity of recalculated nutrient data [4]. They found strong agreement between recalculated and original values for total energy intake, carbohydrate, protein, fat, saturated fat, cholesterol, calcium, sodium, potassium, vitamin C, and vitamin A. Our findings were roughly consistent with Bazzano’s findings for total energy intake, carbohydrate, protein, fat, SFA, and cholesterol.

These updated recalculated nutrient data can be used to address current nutrition issues cost-efficiently, particularly for a long-term follow-up study, such as the 49-year follow-up NHLBI Twin Study. One example is to construct dietary pattern scores such as the Dietary Approaches to Stop Hypertension diet (i.e., DASH diet) and Healthy Eating Index (HEI) to evaluate the whole diet quality from baseline to current day [7,23,27]. Another example is to evaluate the association of a wide range of nutrients and dietary patterns with hard outcomes independent of genetic and environmental factors, which are shared between co-twins including age, cohort, period effects, and secular trend [28].

Expansion of the original NHLBI Twin Study nutrient dataset is theoretically and practically feasible because updated versions of a food composition database usually includes more types of nutrients with their content than earlier ones. SR21, published in 2008, includes the content of 14 types of SFA, 14 types of MUFA, 22 types of PUFA, 13 types of vitamins, and 11 types of minerals [21]. By comparison, the food composition database at the NHLBI 1969 dietary data collection provided no MUFA or SFA fractionation. Data on several nutrients, such as vitamins and minerals, did not exist in the baseline NHLBI dataset. Given the strong agreement between the recalculated and original data in our study and the findings from Bazzano et al. [4], the recalculated data of nutrients that did not exist in the original NHLBI dataset would be expected to be acceptable. Our study also demonstrated that the ranking of participants was not materially affected. Thus, recalculation of intake of nutrients is a very cost-efficient manner for exploratory analyses in relation to long-term outcomes.

There are limitations in our study. Food nutrient and compound contents can change over time. If such changes were reflected in the food composition database, a systematic error might occur for estimating the absolute intake value of a dietary factor. Bazzano et al. pointed out that the recalculated nutrient data might have underestimated the fat content [4]. The empirical rule describes that 95% of observed values lies between mean − 2SD and mean + 2SD and 5% of observations should fall in the range outside of the range of mean ± 2SD [29,30]. Our results were consistent with the empirical rule. For all nutrients, less than 5% of their values were outside of the range of mean ± 2SD. It was, therefore, acceptable [31].

Nutrients and dietary compounds derived from a food composition database, the traditional method, might be more subjective than nutrient biomarkers, such as nutrient concentrations or their metabolites in the blood. However, dietary biomarkers could be influenced by individual variations in metabolism (including catabolism, anabolism, and interactions among dietary compounds), physiological needs, the in vivo storage status of the dietary compounds, and thus may reflect the level of dietary compounds after absorption and metabolism rather than their habitual dietary intake [32,33]. It is extremely costly to measure biomarkers of all nutrients and compounds in the food composition database in a large-scale population study. The biospecimens may be limited for measuring all nutrients and compounds. Dietary compounds in the stored biospecimen may be degraded in the long-term prospective study. Therefore, calculated nutrient and dietary compound intake are still pivotal in investigating the role of diet in disease.

This study has several advantages. Use of the recalculation method to expand the dietary factor dataset is obviously inexpensive and efficient [34,35]. In our study, several different statistical methods were employed to comprehensively evaluate agreement between the recalculated dataset and the original one; our results were robust.

## 5. Conclusions

In conclusion, using reliable means, a recalculated nutrient intake can be used to evaluate associations among dietary compounds and long-term outcomes. Our study demonstrated a very strong agreement between the recalculated and the original nutrient intake, supporting reliability for this method. We also illustrated an efficient way to recalculate the NHLBI Twin Study database. This approach provides opportunity to study the influence of nutrition on cardiovascular disease in breadth and depth. It is feasible to apply this method for recalculating other nutrient datasets for epidemiological studies.

## Figures and Tables

**Table 1:** Food items in the NHLBI Twin Study food composition Table 3.3.0.1 used for SR21.

whole milk, skim milk, tea, coke/soft drink, coffee, cheese other than cottage cheese, ice cream, sweet rolls, cake/pie, eggs, salads, potatoes, cooked vegetables, spaghetti, rice, cereals, fruit juice, fruit, gravy, jam, peanut butter, beer, wine, alcohol (distilled), pork, beef, hamburger, hot dog/luncheon meats, chicken/turkey, lamb, liver, shellfish, other fishes, oil for fried food, chocolate, candy (hard), nuts, potato chips, bread, butter, sugar added in coffee, cream added in coffee, milk added in coffee, cream and sugar added in coffee, milk and sugar added in coffee, sugar added in tea, cream added in tea, milk added in tea, cream and sugar added in tea, milk and sugar added in tea, oil and vinegar type salad dressing, mayonnaise, cheese-type salad dressing

Note: please contact the corresponding author for more detailed information.

**Table 2:** Descriptive analyses for recalculated and original nutrient intake data (*n* = 910).

Nutrient	Estimated Intake Per Day		Paired Difference ^1^	Percent Mean Difference ^2^ (%)
	Recalculated	Original		
	Mean ± SD ^3^	Mean ± SD	Mean ± SD	
Energy (kcal/day)	2051 ± 589	2022 ± 626	28.5 ± 187	2.5
Total carbohydrates (g/day)	224 ± 73.4	225 ± 75.5	−1.6 ± 19.6	−0.3
Protein (g/day)	72.3 ± 20.3	75.1 ± 21.9	−2.7 ± 4.9	−3.1
Fat (g/day)	87.5 ± 32.2	91.2 ± 34.5	−3.7 ± 6.1	−3.7
Saturated fat (g/day)	36.6 ± 14.8	35.7 ± 14.2	0.9 ± 3.9	2.7
Polyunsaturated fat (g/day)	11.7 ± 4.4	8.0 ± 4.2	3.7 ± 2.7	62.6
Monounsaturated fat (g/day)	30.1 ± 11.1	47.5 ± 18.2	−17.5 ± 7.8	−36.3
Cholesterol (mg/day)	436 ± 246	484 ± 314	−47.2 ± 80.5	−5.9

^1^ Paired difference = recalculated value minus original value; ^2^ Percent mean difference = paired mean difference divided by the original mean value; ^3^ SD: Standard deviation.

**Table 3:** Correlation Coefficients and linear regression analyses of recomputed nutrient intakes with those in the original dataset (*n* = 910).

Nutrient	Correlation Coefficients			Regression Analysis	
	Intra-Class		Pearson’s	*R*-Square	β Coefficient (95% CI)
	ICC	(95% CI)			
Energy (kcal/day)	1.00	(1.00, 1.00)	0.95 ^1^	0.91	1.01 (0.99, 1.04)
Total carbohydrates (g/day)	1.00	(1.00, 1.00)	0.97 ^1^	0.93	0.99 (0.98, 1.01)
Protein (g/day)	1.00	(1.00, 1.00)	0.98 ^1^	0.95	1.05 (1.04, 1.07)
Fat (g/day)	1.00	(1.00, 1.00)	0.99 ^1^	0.97	1.06 (1.04, 1.07)
Saturated fat (g/day)	1.00	(1.00, 1.00)	0.96 ^1^	0.93	0.92 (0.90, 0.94)
Polyunsaturated fat (g/day)	1.00	(1.00, 1.00)	0.80 ^1^	0.63	0.76 (0.73, 0.80)
Monounsaturated fat (g/day)	1.00	(1.00, 1.00)	0.97 ^1^	0.95	1.59 (1.57, 1.62)
Cholesterol (mg/day)	1.00	(1.00, 1.00)	0.99 ^1^	0.98	1.26 (1.25, 1.27)

^1^*p*-value < 0.05.

**Table 4:** Quintile agreement and extreme quintile disagreement percent between recomputed and original nutrient intakes (*n* = 910).

Nutrient	Same Quintile (%)	Opposite Extreme Quintile (%)
Energy (kcal/day)	78.6	0.1
Total carbohydrates (g/day)	92.0	0.1
Protein (g/day)	80.2	0
Fat (g/day)	90.7	0
Saturated fat (g/day)	75.4	0.1
Polyunsaturated fat (g/day)	51.2	0.1
Monounsaturated fat (g/day)	80.3	0
Cholesterol (mg/day)	79.7	0
Mean of percentage	78.5	0.1

## Abbreviations

CI	confidence intervals
MUFA	monounsaturated fatty acids
NHLBI	National Heart, Lung, and Blood Institute
PUFA	polyunsaturated fatty acids
*R* ^2^	*R*-square
SD	standard deviations
SFA	saturated fatty acids
SR21	Standard Reference 21
USDA	United States Department of Agriculture

## References

[1] Coulston A.M., Boushey C., Ferruzzi M.G., Delahanty L.M. (2017). Nutrition in the Prevention and Treatment of Disease.

[2] Shim J.S., Oh K., Kim H.C. (2014). Dietary assessment methods in epidemiologic studies. Epidemiol. Health.

[3] Greenfield H., Southgate D.A.T. (2003). Food Composition Data: Production, Management and Use.

[4] Bazzano L.A., He J., Ogden L.G., Loria C.M., Vupputuri S., Myers L., Whelton P.K. (2002). Agreement on nutrient intake between the databases of the first national health and nutrition examination survey and the ESHA food processor. Am. J. Epidemiol..

[5] Hjortland M. (1972). The effects of heredity and environment on nutrient intake of adult monozygotic and dizygotic twins. Ph.D. Thesis.

[6] Fabsitz R.R., Garrison R.J., Feinleib M., Hjortland M. (1978). A twin analysis of dietary intake: Evidence for a need to control for possible environmental differences in MZ and DZ twins. Behav. Genet..

[7] Dai J., Krasnow R.E., Reed T. (2016). Midlife moderation-quantified healthy diet and 40-year mortality risk from CHD: The prospective National Heart, Lung, and Blood Institute Twin Study. Br. J. Nutr..

[8] Kandula N.R., Puri-Taneja A., Victorson D.E., Dave S.S., Kanaya A.M., Huffman M.D. (2016). Mediators of Atherosclerosis in South Asians Living in America: Use of Web-Based Methods for Follow-Up and Collection of Patient-Reported Outcome Measures. JMIR Res. Protoc..

[9] Woynaroski T., Oller D.K., Keceli-Kaysili B., Xu D., Richards J.A., Gilkerson J., Gray S., Yoder P. (2017). The stability and validity of automated vocal analysis in preverbal preschoolers with autism spectrum disorder. Autism Res. Off. J. Int. Soc. Autism Res..

[10] Elmadfa I., Meyer A.L. (2010). Importance of food composition data to nutrition and public health. Eur. J. Clin. Nutr..

[11] Wolmarans P., Kunneke E., Laubscher R. (2009). The use of the South African Food Composition Database System (SAFOODS) and its products in assessing dietary intake data. Part II. S. Afr. J. Clin. Nutr..

[12] Dresser C.M. From nutrient data to a data base for a health and nutrition examination survey. Organization, coding and values-real or imputed. Proceedings of the 8th National Nutrient Data Base Conference.

[13] Dawber T.R., Pearson G., Mann G.V., Kannel W.B., Shurtleff D., McNamara P. (1962). Dietary assessment in the epidemiologic study of coronary heart disease: The Framingham study. II. Reliability of measurement. Am. J. Clin. Nutr..

[14] Moghames P., Hammami N., Hwalla N., Yazbeck N., Shoaib H., Nasreddine L., Naja F. (2016). Validity and reliability of a food frequency questionnaire to estimate dietary intake among Lebanese children. Nutr. J..

[15] Dai J., Krasnow R.E., Liu L., Sawada S.G., Reed T. (2013). The association between postload plasma glucose levels and 38-year mortality risk of coronary heart disease: The prospective NHLBI twin study. PLoS ONE.

[16] Reed T., Carmelli D., Christian J.C., Selby J.V., Fabsitz R.R. (1993). The NHLBI male veteran twin study data. Genet. Epidemiol..

[17] Carmelli D., Swan G.E., Robinette D., Fabsitz R. (1992). Genetic influence on smoking—A study of male twins. N. Engl. J. Med..

[18] Feinleib M., Christian J.C., Borhani N.O., Rosenman R., Garrison R.J., Wagner J., Kannel W.B., Hrubec Z., Schwartz J.T. (1976). National heart and lung institute twin study of cardiovascular disease risk factors—Organization and methodology. Acta Genet. Med. Gemellol..

[19] Burke B. (1947). The dietary history as a tool in research. J. Am. Diet. Assoc..

[20] Mann G.V., Pearson G., Gordon T., Dawber T.R., Lyell L., Shurtleff D. (1962). Diet and cardiovascular disease in the Framingham study. Am. J. Clin. Nutr..

[21] U.S. Department of Agriculture, Agricultural Research Service (2008). USDA National Nutrient Database for Standard Reference, Release 21. Nutrient Data Laboratory Home Page. https://www.ars.usda.gov/northeast-area/beltsville-md-bhnrc/beltsville-human-nutrition-research-center/nutrient-data-laboratory/docs/sr21-home-page/.

[22] Bukenya R., Ahmed A., Andrade J.M., Grigsby-Toussaint D.S., Muyonga J., Andrade J.E. (2017). Validity and Reliability of General Nutrition Knowledge Questionnaire for Adults in Uganda. Nutrients.

[23] Yuan Y.Q., Li F., Wu H., Wang Y.C., Chen J.S., He G.S., Li S.G., Chen B. (2018). Evaluation of the Validity and Reliability of the Chinese Healthy Eating Index. Nutrients.

[24] Daniel W. (1999). Biostatistics: A Foundation for Analysis in the Health Sciences.

[25] Bland J.M., Altman D.G. (2010). Statistical methods for assessing agreement between two methods of clinical measurement. Int. J. Nurs. Stud..

[26] Carithers T., Talegawkar S., Rowser M., Henry O., Dubbert P., Bogle M., Taylor H.J., Tucker K. (2009). Validity and Calibration of Food Frequency Questionnaires Used with African-American Adults in the Jackson Heart Study. J. Am. Diet. Assoc..

[27] Chiu S., Bergeron N., Williams P.T., Bray G.A., Sutherland B., Krauss R.M. (2016). Comparison of the DASH (Dietary Approaches to Stop Hypertension) diet and a higher-fat DASH diet on blood pressure and lipids and lipoproteins: A randomized controlled trial. Am. J. Clin. Nutr..

[28] Liu J., Tuvblad C., Raine A., Baker L. (2013). Genetic and environmental influences on nutrient intake. Genes Nutr..

[29] Liu K., Zhang J., Fu B., Xie H., Wang Y., Qian X. (2014). Evaluation of empirical rule of linearly correlated peptide selection (ERLPS) for proteotypic peptide-based quantitative proteomics. Proteomics.

[30] Grafarend E.W., Awange J.L. (2012). Applications of Linear and Nonlinear Models: Fixed Effects, Random Effects, and Total Least Squares.

[31] Giavarina D. (2015). Understanding Bland Altman analysis. Biochem. Med..

[32] Fairweather-Tait S.J. (1996). Bioavailability of dietary minerals. Biochem. Soc. Trans..

[33] Sugiura S.H., Dong F.M., Rathbone C.K., Hardy R.W. (1998). Apparent protein digestibility and mineral availabilities in various feed ingredients for salmonid feeds. Aquaculture.

[34] Holden J.M., Eldridge A.L., Beecher G.R., Buzzard I.M., Bhagwat S., Davis C.S., Douglass L.W., Gebhardt S., Haytowitz D., Schakel S. (1999). Carotenoid content of U.S. foods: An update of the database. J. Food Comp. Anal..

[35] Willett W. (1998). Nutritional Epidemiology.

